# Circular RNAs Are the Predominant Transcript Isoform from Hundreds of Human Genes in Diverse Cell Types

**DOI:** 10.1371/journal.pone.0030733

**Published:** 2012-02-01

**Authors:** Julia Salzman, Charles Gawad, Peter Lincoln Wang, Norman Lacayo, Patrick O. Brown

**Affiliations:** 1 Department of Biochemistry, Stanford University School of Medicine, Stanford, California, United States of America; 2 Howard Hughes Medical Institute, Stanford University School of Medicine, Stanford, California, United States of America; 3 Department of Pediatric Hematology/Oncology, Stanford University School of Medicine, Stanford, California, United States of America; The John Curtin School of Medical Research, Australia

## Abstract

Most human pre-mRNAs are spliced into linear molecules that retain the exon order defined by the genomic sequence. By deep sequencing of RNA from a variety of normal and malignant human cells, we found RNA transcripts from many human genes in which the exons were arranged in a non-canonical order. Statistical estimates and biochemical assays provided strong evidence that a substantial fraction of the spliced transcripts from hundreds of genes are circular RNAs. Our results suggest that a non-canonical mode of RNA splicing, resulting in a circular RNA isoform, is a general feature of the gene expression program in human cells.

## Introduction

Deep sequencing of RNA from biological samples, “RNA-Seq”, is a powerful tool for discovering and cataloguing novel alterations in the expression, sequence, and structure of transcriptomes. In the present study, we used RNA-Seq in a deliberate search for transcripts that could not be accounted for by conventional splicing of primary transcripts from an unrearranged human genome. Although our initial goal was to discover cancer-specific chromosomal rearrangements by identifying the resulting fused or rearranged transcripts, we also investigated the possibility that the exon order specified by the genome sequence might be rearranged during RNA processing. We were surprised to find numerous examples of transcripts in which the exon order was a circular permutation of the order encoded in the genome. We hypothesized that these anomalous transcripts might the result of intramolecular but non-canonical splicing events that joined a splice donor to an upstream (i.e. toward the 5′ end of the transcript) splice acceptor to produce a circular RNA molecule. Indeed, for many genes, in both cancer and normal human cells, we found RNAs with circularly permuted exon orders at levels comparable to those of the canonical, linear mRNA.

The first observation suggesting that eukaryotic RNAs can exist in circular form was made more than 30 years ago by electron microscopy [1]. 10 years later, human cytoplasmic RNA was reported to contain very low levels of transcripts of the DCC gene with exons spliced in non-canonical order (i.e. shuffled relative to the reference genome). These scrambled transcripts were estimated to comprise less than one one-thousandth of DCC transcripts, and the phenomenon was dubbed exon-scrambling [2]. Since that time, a handful of expressed mammalian genes have been shown to express circular RNA isoforms at low levels [3], [4]. Such examples include very low levels of human RNA transcripts with scrambled exons observed in several human genes, including MLL and ETS-1 [13], [14].

The best-characterized circular transcripts are in rodents. The mouse SRY gene, the sex-determining gene in males, consists of a single exon. During development, the RNA exists as a linear transcript that is translated into protein. In the adult testes, the RNA exists primarily as a circular product that is predominantly localized to the cytoplasm and is apparently not translated [5], [6]. Studies have demonstrated that inverted repeats in the genomic sequence flanking the SRY exon direct transcript circularization [5], [7], [8]. The sodium transporter NCX1 and the rat cytochrome P450 2C24 gene are two other well-studied examples of mouse transcripts with circular isoforms that are expressed at relatively low levels [9]–[11]. The circular isoform of the NCX1 gene is thought to encode a protein, although this possibility has not been conclusively demonstrated. Examples of exon scrambling have also been found in Drosophila [12].

All examples of circular transcripts reported to date in humans have been found to be expressed at low levels compared to the dominant canonical linear isoform, requiring sensitive nested PCR experiments for detection; these examples were discovered inadvertently or in an effort to characterize the structure of oncogenes. Circular RNAs have also been reported to be rare isoforms of the human Cytochrome P-450 2C18, and dystrophin transcripts [11], [15]. Most recently, a circular isoform of the non-coding RNA ANRIL was found to be expressed at very low levels; its expression was correlated with INK4/ARF expression, and with risk for developing atherosclerosis [16]. This finding suggests that circular RNAs may have both biochemical and phenotypic consequences. Because of their apparent rarity, however, circular RNAs are generally viewed as minor RNA structural variants, perhaps attributable to transcriptional noise [17].

Two studies have suggested that scrambled exons may occur in more than a handful of transcripts. Roughly 150 sequences representing exon scrambling were found in EST databases; in each case, the authors argued that these sequences were unlikely to derive from circular RNA molecules, but rather represented bona fide examples of exon scrambling within a linear transcript [18], [19]. The lack of direct experimental evidence makes interpretation of these studies difficult: EST database studies are fraught with confounding of RT-PCR artifacts and selection bias. If template switching occurs during reverse transcription, given a typical human exon length of 150 (the average of the lower 90th quantile), 1 out of 22500 template switches would be at exon-exon boundaries, and hundreds could be expected to be detected in an EST database. Indeed, it has been recently suggested that apparent examples of exon scrambling in human EST databases are primarily explained as RT artifacts. More recently, a single-read RNA-Seq study of poly-adenylated human RNA from a wide variety of human tissues identified 176 genes with 205 scrambled isoforms, achieving a 64% validation rate with RT-PCR, and showing that many such transcripts had high expression levels compared to the canonical transcripts [20]. These authors postulated that the RNA molecules containing scrambled exons were linear and poly-adenylated.

## Results

### Hundreds of Putative Intragenic Rearrangements in Pediatric Acute Lymphoblastic Leukemia

We recently reported evidence suggesting that local structural rearrangements in the human genome may be under-recognized genetic lesions contributing to the progression of human cancers [21]. To test this hypothesis, we explored whether intragenic rearrangements were prevalent in childhood cases of Acute Lymphoblastic Leukemia (ALL) by performing RNA-Seq on ribosomal RNA-depleted total RNA from the diagnostic bone marrow of 5 children between the ages of 2 and 6 with hyperdiploid B-precursor ALL. We searched for transcripts with scrambled exon order (i.e. where exon X is upstream of exon Y, but X≥Y) that might represent intragenic rearrangements by comparing paired-end RNA-seq data with a database of human transcriptome sequences (UCSC knowngene hg19). Reads that could not be aligned to the UCSC knowngene transcriptome were subsequently aligned to a custom database of exon-exon junctions comprising all possible pairs of exons annotated in hg19 RefSeq. A paired-end read is considered ‘junctional’ if read 1 aligns to the gene and read 2 aligns to an exon-exon junction with more than 10 mismatch-free bases flanking each side of the junction. The essential features of this analysis are described in the methods section and have been described in greater detail previously [21].

This analysis identified hundreds of genes with evidence of exon scrambling: in all, more than 1232 such genes had more than one junctional read. In addition, more than 700 isoforms with scrambled exons were estimated to comprise more than 10% of all transcript isoforms for a comparable number of genes. Genes with evidence of scrambling were shown to be statistically shared between the three leukocyte populations. A complete list of scrambled isoforms is provided in Table S1. The most abundant transcripts with scrambled exons were ESYT2, FBXW4, CAMSAP1, KIAA0368, CLNS1A, FAM120A, MAP3K1, ZKSCAN1, MANBA, ZBTB46, NUP54, RARS, and MGA. Each was confirmed by RT-PCR.

### Exon Scrambling in Normal Human Tissues

**Figure 1:**
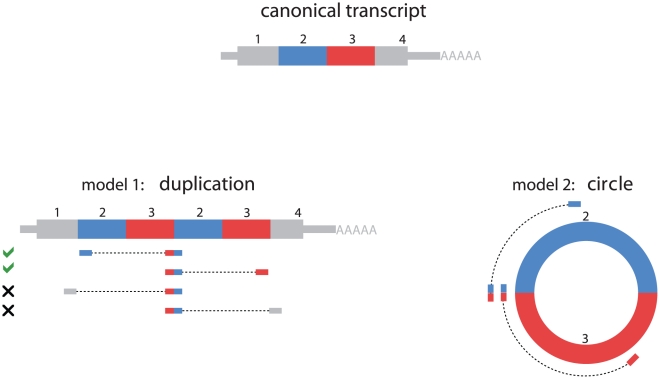
Models to explain exon scrambling. The canonical linear reference transcript is depicted with exons as colored boxes with four exons 1, 2, 3, and 4. Two simple models of RNA structure that could explain scrambled transcripts are depicted at left and right. At left, model 1 depicts how a scrambled exon 3-exon 2 junction could arise from a tandem duplication of exons 3 and 2, positioning the first copy of exon 3 upstream of exon 2. At the RNA level, this event could arise from post-transcriptional exon rearrangement, or a genomic duplication of exons 2 and 3. Under the model of tandem duplication, when one side of a paired-end read maps to the junction between exon 3 and 2, the other may map to any of exons 1, 2, 3 or 4 with probabilities determined by the library's insert length distribution and the exon lengths. Our data supports paired-end mapping between a junction and exons 2 or 3, but not exons 1 and 4. We note that in principle, the scrambled exon 3 - exon 2 junction could arise from other splicing events and does not necessarily entail tandem duplication. At right, model 2 depicts how a scrambled exon 3 - exon 2 junction could arise from splicing of exons 2 and 3 into a circular RNA molecule, again positioning exon 3 upstream of exon 2. In this model, when one side of a paired-end read maps to the junction between exon 3 and 2, the other will map to exon 2 or exon 3.

Two models for the origin of the scrambled exons are diagrammed in Figure 1. We began with the hypothesis that scrambled exons would generally be a sign of local genomic rearrangements in cancer. To our surprise, all examples of exon scrambling that we PCR-verified in leukemia samples were also detectable in HeLa cells and normal primary human cells, including peripheral blood collected from the same ALL patients in remission and H9 ES Cells. These results strongly suggested that the vast majority of scrambled exons detected in tumor samples were consequences of splicing processes active in both normal and malignant human cell types.

We therefore analyzed RNA from 3 leukocyte cell types: naïve B cells (CD19+), hematopoietic stem cells (CD34+) and neutrophils from the same individual, for the presence of scrambled exons, by paired-end sequencing (80 bp per end) of ribosomal RNA-depleted (Ribo-zero) total RNA. We obtained roughly 8, 19, and 5 million reads aligning to the UCSC knowngene transcriptome (mapped as single reads) from CD19+, CD34+, and neutrophils, respectively. We found sequence evidence for scrambled exons comprising at least 10% of transcripts from each of more than 800 genes in CD19+ cells, CD34+ cells, and neutrophils, respectively. Genes with evidence of scrambling in one leukocyte cell type were significantly more likely also to have transcripts with scrambled exons in one or more of the other cell types we analyzed.

We also analyzed publicly available paired-end data from poly-A selected HeLa and H9 Human embryonic stem cells [22] using the same pipeline described above. In total, we identified 2748 transcript isoforms with scrambled exons; some of these isoforms were rare compared to their canonical linear counterparts, but for a large number of genes, circular isoforms comprised a substantial fraction of all transcripts (see Table S2 for a complete list of scrambled exons detected).

The scrambled exon junctions in transcripts of C1orf58, CCDC126, FKBP8, PCMTD1, SMARCA5, and SETD2 predicted by the primary sequence data, were all directly confirmed by RT-PCR from normal leukocyte RNA, followed by Sanger sequencing. Eleven additional instances of exon scrambling in eight distinct genes in ALL or HeLa were also confirmed by RT-PCR and Sanger Sequencing (see Table S3).

### Scrambled Isoforms of Many Transcripts Are Expressed at Levels Comparable to the Canonical Linear Isoforms

**Figure 2:**
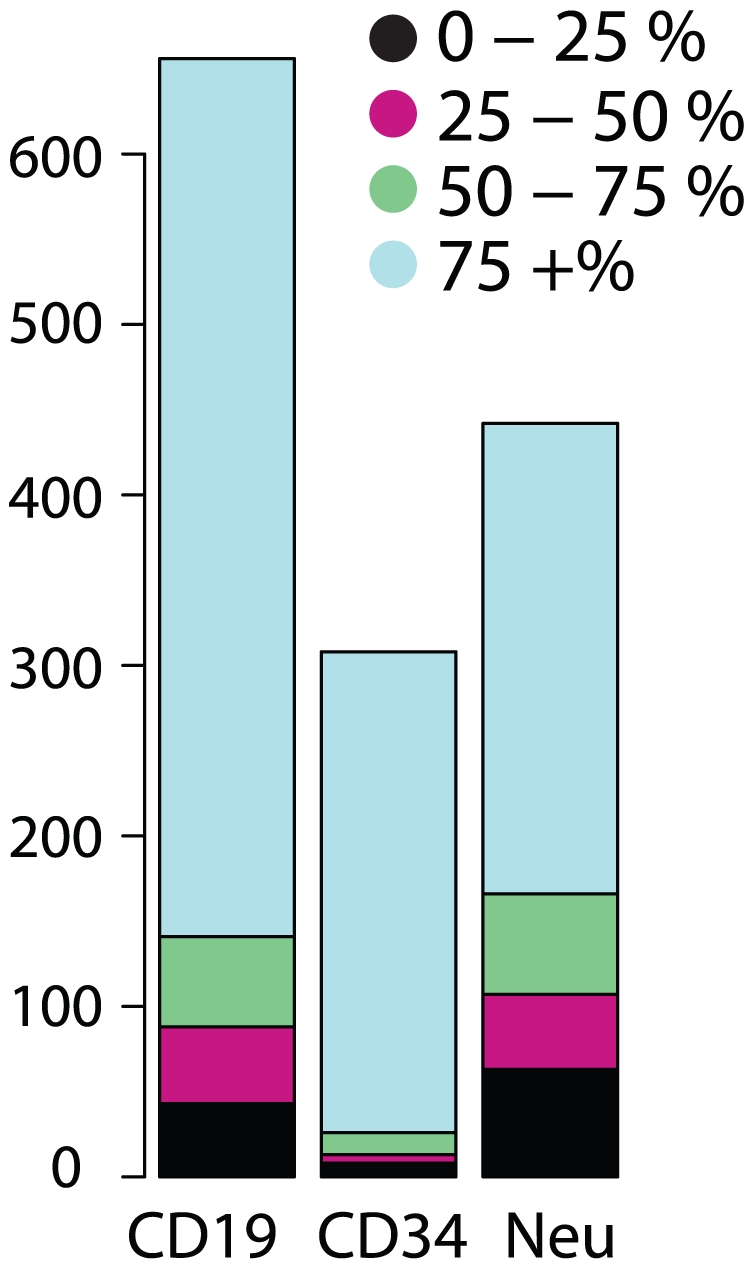
Expression levels of scrambled exons. Analysis of paired-end RNA-Seq data from random primed libraries reveals evidence that scrambled exons are present at high stoichiometries compared to the canonical linear transcript transcribed from a large number of human genes. This phenomenon persists across cell types and is illustrated by the expression patterns of 3 leukocyte cell types: CD19 (B cells), CD34 (stem cells) and neutrophils. The fraction of each scrambled transcript as a fraction of total gene expression is computed. The bar plot depicts the number of circular isoforms with estimated abundance relative to all transcripts of the gene in the following ranges: between 0–25%, 25–50%, 50–75% and 75+%. Hundreds of isoforms in each cell type are estimated to represent more than half of all transcripts from each gene.

We estimated the abundance of scrambled isoforms relative to their canonical linear counterparts by two genome-wide statistical methods. For the first estimate, we counted paired-end reads in which one read mapped to a scrambled junction (a junction between exon-X and exon-Y where X≥Y), and the other read also mapped in an orientation and position consistent with the hypothetical scrambled transcript. This number was then compared to the total number of paired-end reads consistent with the splice junctions between exon X and exon Y in the canonical order predicted by the reference genome sequence. The relative abundance of scrambled transcript isoforms as a fraction of all transcripts for a given gene is depicted for three normal leukocyte cell types in Figure 2. Note that assuming sequencing reads are sampled uniformly across each transcript, this method underestimates the total fraction of transcripts containing exons in scrambled order, as scrambled transcripts are also expected to yield read pairs corresponding to the canonical linear orientation.

By this first method (method 1), we identified 229, 207, and 122 scrambled transcripts, representing 481 distinct genes, that were expressed at levels comparable to the canonical linear isoforms in human CD19+ leukocytes, CD34+ leukocytes and neutrophils, respectively. By the same method, we estimated that 513, 275, and 320 scrambled transcripts, representing 880 distinct genes, were at least 10% of the abundance of the canonical isoform, in CD19+ cells, CD34+ cells and neutrophils, respectively. The differences among these cell types in the apparent relative abundance of the scrambled isoform were consistent with sampling variation due to differential expression of the corresponding gene among these cell types (see Text S1).

Our direct observations of scrambled isoforms indicate that they comprise at least 10% of all transcripts for roughly 1–2% of genes detectably expressed in the CD19+ cells, CD34+ cells and neutrophils. Ignoring relative expression level of the scrambled isoform, we identified a scrambled isoform in roughly 10% of all detectably expressed transcripts. This is likely to be an underestimate of the prevalence of scrambled isoforms, as the pathognomonic junctional reads from inabundantly expressed genes were unlikely to be detected with this sequencing depth - more than half of the distinct transcript isoforms detected in these samples were sequenced at an average depth of one or less (Figure S1).

The most abundant transcripts with scrambled exon order in the CD19+, CD34+, neutrophils samples, respectively, corresponded to KIAA0182 (encoding a putative subunit of the BRAF-HDAC complex), MAN1A2 (encoding an alpha-mannosidase), and CCDC126 (encoding a coiled-coiled containing protein of unknown function). Exon-scrambled isoforms accounted for more than half of all transcripts from each of these genes, as estimated by method 1 above. We also detected low levels of scrambled isoforms of noncoding RNAs (see NR identifiers in Tables S1, S2).

In addition, we used a second statistical method that did not rely on exon annotation to detect and quantitate transcripts with scrambled exons. For each 200 bp window tiling the length of each gene, we compared the number of paired-end reads in two categories; category 1, where side 1 mapped to a fixed window and side 2 mapped in a relative orientation to side 1 inconsistent with the genomic exon order; and category 2, paired-end reads where side 1 mapped to a window with a relative orientation to side 2 consistent with the genomic order. This procedure allowed us to identify candidate exon-scrambled transcripts without relying on a database of hypothetical scrambled exon junction. Table S4 presents these results for the leukemia data. We tested one gene, FBXW7, a well-known tumor suppressor with strong evidence of exon scrambling determined by this approach, which did not have evidence of scrambling from a custom exon-exon junction database. Using outward primers in the second coding exon of the RefSeq annotation, we detected scrambling between the second annotated exon in the RefSeq database for FBXW7 and an alternative 5′ UTR exon present only in the UCSC known-gene annotation. We verified the scrambled exon-exon junction by Sanger Sequencing of the product amplified from HeLa cDNA (see Table S3); it was also detected in ALL and H9 samples.

### Statistical Tests Predict that Most Scrambled Exons Are Due to Circular Transcripts

We considered two models to explain the observed transcripts with scrambled exon order (Figure 1): 1. A tandem duplication of one or more contiguous exons in canonical order (the tandem duplication hypothesis), due either to a genomic rearrangement resulting in an intragenic duplication, or trans-splicing; 2. A circular RNA formed by splicing a splice donor from a downstream exon to a splice acceptor from an upstream exon (the circular RNA hypothesis). The two models predict significantly different distributions of paired-end sequences when the size of the hypothetical circular form is comparable to the insert length in the library we used for sequencing (roughly 300–500 bp with tails extending roughly 100 bp).

In cases where the size of the hypothetical circular isoform was comparable that of the inserts in our library, we used a statistical approach to evaluate the evidence that observed scrambled exons originated in a circular RNA. To illustrate this method, a junctional read supporting the presence of exon 3 upstream of exon 2 could be explained by a 2-exon circle composed of exons 2 and 3, or a tandem duplication of exons 2 and 3. Only in the latter case would we expect to observe paired reads comprising the non-canonical 2–3 junction paired with a sequence from exon 1 or exon 4.

Reads supporting the tandem duplication hypothesis provide direct evidence that only a very small fraction of transcripts with scrambled exons are consistent with a linear RNA structure: in our sequence data from human leukocytes we found evidence of a linear RNA with scrambled exons for only 23 genes, fewer than 2% of all transcripts with evidence of scrambled exons (see Table S6). The genes for which we found such direct evidence of linear transcripts with scrambled exons were not, on average, more highly expressed than genes with evidence of circular RNAs, suggesting that inadequate sampling depth does not account for their relative rarity. When for a given gene we could find no paired-end sequence evidence for a tandem duplication– the vast majority of cases– we used statistical inference to assess the strength of this evidence against a tandem duplication by leveraging the insert length distribution and the size of the inferred circular RNA. A genome-wide assessment of this evidence in the leukocyte data provides compelling evidence against tandem duplications in the vast majority of transcripts (see Table S5).

About 32% of the scrambled exon junctions we observed were consistent with a hypothetical circular RNA larger than the insert lengths that were well-sampled in our library; in such cases, the statistical approach described above could not be used to infer the probable structure of these isoforms. However, as detailed below, biochemical analysis of nine isoforms with scrambled exons, including cases in which the inferred size of the hypothetical circular RNA would be greater than the insert length distribution, provided strong evidence that most scrambled exons represent circular RNA molecules.

### RNA Isoforms with Scrambled Exons Are Circular

**Figure 3:**
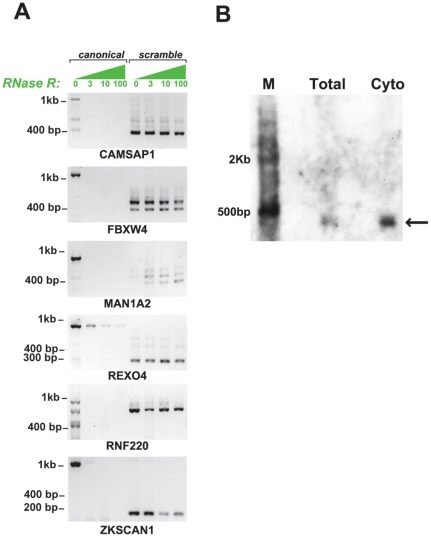
RNaseR assay confirms scrambled exons arise from circular RNA. Panel A: Total RNA from HeLa cells was digested with RNaseR at varying enzyme concentrations (0, 3, 10, and 100 units) after the RNA was depleted of ribosomal RNA. Primers capable of amplifying the canonical linear transcript and the predicted circular transcript (by outward facing primers within a single exon predicted in the scramble) were used in a RT-PCR experiment for each of the digestion conditions. Canonical transcripts were consistently degraded by RNaseR, only detectable by PCR at 0 units of RNaseR, whereas predicted circular transcripts consistently resisted the RNaseR challenge, providing strong evidence of circularity. FBXW4 and MAN1A2 respectively show 2 and 4 circular isoforms, both of which were predicted by the sequencing data. The predicted lengths of circular isoforms are respectively a 3-2 junction of CAMSAP1 (predicted to produce a 435 bp circle), a 4-2 and 5-2 junction of FBXW4 (predicted to produce 415 and 510 bp circles), a 4-2, 5-2 and 6-2 junction of MAN1A2 (predicted to produce 471, 553, and 648 bp circles), a 3-3 junction in REXO4 (predicted to produce a 338 bp circle), a 2-2 junction of RNF220 (predicted to produce a 742 bp circle) and a 3-2 junction of ZKSCAN1 (predicted to produce a 667 bp circle). Panel B: A northern blot on total and cytoplasmic lysate from HeLa cells shows hybridization of a 481 bp probe complementary to the MAN1A2 5-2 exon scramble. 3.7 and 6.2 ug of total and cytoplasmic RNA were loaded onto a 1% agarose gel and 10 pM of probe was hybridized for 24–48 hours. Detection was performed using the BrightStar BioDetect Kit (Ambion, Austin, TX). The specific band at 553 bp corresponds to the predicted size of a circular RNA containing exons 2,3,4 and 5 of MAN1A2.

We used susceptibility to RNaseR as a biochemical assay for the topology of the RNA molecules containing scrambled exons. RNaseR is a 3′ to 5′ exoribonuclease that degrades essentially all linear RNA molecules, regardless of structure, provided the RNA contains a 7 bp 3′ overhang [23]. To optimize the efficiency of RNaseR digestion, HeLa RNA was first depleted of ribosomal RNA and then subjected to RNaseR digestion. We tested a panel of 6 genes, including 9 isoforms with scrambled exons for sensitivity to RNaseR. The inferred size of the hypothetical circular RNAs that could account for the exon scrambles we sampled spanned a wide range, including sizes larger than the maximum insert lengths of our libraries. As predicted from the circular RNA model, each of these transcripts was resistant to RNaseR degradation, suggesting that most scrambled transcripts are circular RNAs (examples shown in Figure 3). In addition, we performed a Northern blot to probe for the 5–2 exon scramble in MAN1A, and determined that the probe specific to the 5–2 junction hybridized to a band matching the size of the predicted RNA circle containing exons 2, 3, 4 and 5 of MAN1A2 (see Figure 3). Control PCR assays omitting either template or RT yielded no product (see Figure S2).

### Evidence that Most Scrambled Exons Are Not Poly-adenylated

Using a publicly available dataset of short sequence reads from matched poly-A selected and poly-A depleted RNA fractions, we further tested the hypotheses that a) a subset of the scrambled exons we identified are contained in linear, poly-adenylated RNA molecules and b) a second subset of the scrambled exons we identified are contained in non-poly-adenylated, presumably circular, molecules. More specifically, the RNA samples from which these sequences derived were obtained by a double poly-A selection; the selected molecules were prepared for sequencing; the flow-through fraction from the second poly-A selection was depleted of ribosomal RNA and also sequenced [22]. In each of the 4 data sets: both poly-A enriched and depleted from both HeLa and H9 ES cell lysates, we counted the number of reads mapping uniquely to any of the scrambled exon-exon junctions that we had previously identified as putative circular RNAs in our own sequencing experiments on leukocytes.

**Figure 4:**
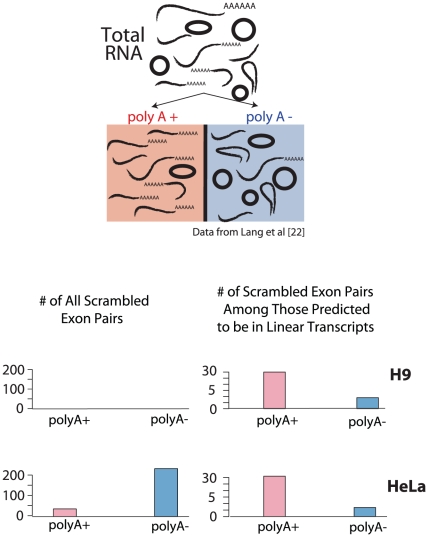
Scrambled exons are enriched in poly-A depleted samples. Single-end 76-bp RNA-Seq was performed on matched experiments on HeLa, and H9 Human embryonic stem cell lysates were polyA selected and polyA depleted (data from Yang et al [22]). The numbers of scrambled exons detected in each sample which appeared in our curated database of scrambled junctions from the leukocyte data are depicted as colored bars. Roughly equal numbers of sequencing reads were available from each of 4 samples. Left panels of bar plot: both H9 and HeLa cells show markedly more exon scrambles in polyA depleted fractions compared to polyA enriched fractions, consistent with scrambles arising from circular transcripts which lack polyA tails. Right panels of bar plot: conversely, in the much smaller subset of scrambled exon pairs where we have evidence of internal tandem duplication (i.e. evidence against circularity), we find the opposite enrichment: more exon scrambles in polyA enriched fractions compared to polyA depleted fractions, consistent with this small subset of scrambles arising from linear, polyA transcripts.

Overall, the scrambled exon junctions from suspected circular RNAs were observed at tenfold higher frequency in the poly-A depleted fraction in both HeLa and H9 cells than in the poly-A selected fractions (see Figure 4). This result further suggests that the majority of high confidence scrambled exon junctions lack poly-A tails, as expected for circularly spliced RNAs. Strikingly, the opposite bias in enrichment was observed for scrambled exon junctions that we had inferred from RNA-Seq evidence to be contained in linear RNA molecules: reads representing transcripts that we predicted to be linear molecules with tandemly duplicated exons were roughly 3-fold more prevalent in the poly-A selected libraries than in the poly-A depleted libraries from both HeLa and H9 cells (see Figure 4).

### Some Scrambled-Exon Transcripts May Provide Evidence for Intragenic Duplications in the Human Genome

For a small fraction of the scrambled exon junctions we found compelling RNA-Seq evidence that they derived from linear RNAs. In one case, the apparently “scrambled exons” are explained by a known exon duplication in the human genome (IFI16) which served as a convenient positive control; in another case (STAU2), the exons affected by the apparent tandem duplication correspond to a known human structural variant (see Table S6 for a complete list derived from poly-A selected RNA from HeLa, H9 cells and leukocytes). While we have not exhaustively tested all scrambled transcripts that are predicted to be linear by RNA-Seq analysis, we believe they will likely provide a source for discovery of structural variants in the human genome that have so far eluded detection by next generation sequencing.

### No Evidence for a Relationship Between Scrambled Exons and Complementary Alternatively Spliced Transcripts

Previous reports have proposed that the lariat structure containing exons excluded by alternative splicing could undergo subsequent splicing to yield circularly spliced isoforms [10], [19]. We therefore searched for evidence of alternatively spliced transcripts that would be predicted to yield an in-frame linear transcript (i.e. one not subject to nonsense-mediated decay) and a lariat containing the exons we found joined in circular RNAs.

Alternatively spliced complements to only 13 of 576 distinct scrambled isoforms were supported by more than 1 sequencing read (2.2%). The failure to detect essentially any evidence for the predicted complementary linear transcripts is circumstantial evidence that most circular transcripts are unlikely to be incidental byproducts of alternative splicing.

In addition to testing for the co-occurrence of complementary alternatively spliced transcripts and circular RNA, we evaluated the organization of genes from which circular transcripts were transcribed. This analysis revealed a statistical enrichment of exon 2 (p value ≪10^−4^) as the acceptor exon in circular RNA, and a statistically significant enrichment in median intron length among genes with circular isoforms, particularly in the intron upstream of acceptor exons and to a lesser extent in the intron downstream of donor exons (see Text S1 and Figure S3).

### Many Circular Transcripts are Cytoplasmic

**Figure 5:**
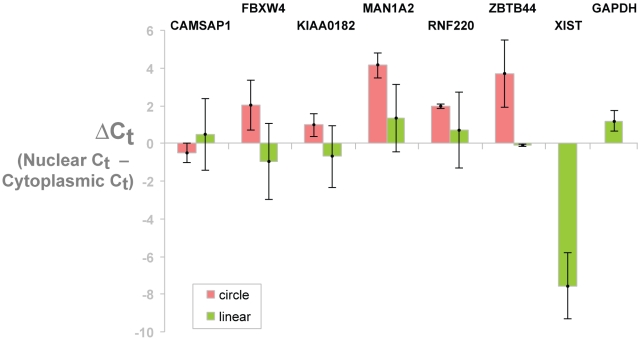
qPCR shows scrambled exons are enriched in the cytoplasm. HeLa whole cells lysates were fractioned into cytoplasmic and nuclear. The nuclear localized noncoding RNA XIST served as a control for fractionation:, and as expected, was enriched in the nuclear fraction. In addition, precursor ribosomal RNA bands were present in the nucleus but not the cytoplasm (see Figure S4). Using probes specific to each canonical and circular isoform (corresponding to those examples depicted in Figure 3), we compared Ct values calculated from qPCR on cDNA from the cytoplasmic fraction to the Ct value from qPCR on cDNA from the nuclear fraction. Bar heights show this average Ct value difference across 2 biological replicates. Error bars represent 2.5 standard deviations computed from biological variation of the qPCR assay. These results show that most circular isoforms are more enriched in the cytoplasm compared to the canonical linear isoforms.

To begin investigating cellular localization of circular transcripts, we studied the localization of an isoform of MAN1A2, a ubiquitous and highly expressed circular transcript, and a handful of other isoforms with scrambled exons that we confirmed were circular. HeLa whole cells lysates were fractioned into nuclear and cytoplasmic fractions. The nuclear-localized noncoding RNA, XIST, served as a control for fractionation: as expected, it was enriched in the nuclear fraction and depleted in the cytoplasmic fraction. The total, cytoplasmic, and nuclear RNA were run on a denaturing gel to evaluate the ribosomal RNA components. As expected, the nuclear fraction had diminished 28S and 18S rRNA bands, and ribosomal RNA precursor bands were present in the nuclear, but not the cytoplasmic fraction (see Figure 5 and Figure S4). For each isoform, including XIST, we compared Ct values calculated from qPCR on cDNA from the cytoplasmic fraction to the Ct value from qPCR on cDNA from the nuclear fraction. Technical and biological replicates confirmed that most circular isoforms were, surprisingly, more enriched in the cytoplasmic fraction than were the canonical linear isoforms (see Figure 5).

### Sequencing of Ribosomal RNA-Depleted RNA from Mouse Brain Reveals Hundreds of Scrambled Exon Pairs

We hypothesized that circular RNA isoforms were not uniquely prevalent in human cells, but a common feature of the gene expression program in animals and perhaps more broadly. We analyzed publicly available paired-end RNA-Seq data from ribosomal RNA-depleted total RNA, consisting of 51-bp paired-end reads (id SRR029642 in the short read archive). We analyzed these data using the same pipeline we had applied to the leukocyte data (using the mm9 RefSeq and UCSC annotations (downloaded 9/2011) as the reference). We found more than one thousand mouse genes with evidence of exon scrambling (see Table S8).

## Discussion

For more than 30 years, sporadic reports have described the presence of circular mRNA transcripts in mammals. Many of these circular RNAs were discovered serendipitously, and were largely disregarded as nonspecific byproducts when they were found to be expressed at low levels. In contrast to this prevailing view on the abundance of circular RNA isoforms, we found strong evidence that circular isoforms of hundreds of human transcripts, with out of order splice junctions precisely at normal exon boundaries, were present at levels comparable to their canonical linear counterparts. While the canonical linear transcripts were sensitive to RNaseR treatment in all tested cases, transcripts with scrambled exons were generally resistant to RNaseR treatment, as expected for circular RNAs, but not for RNAs with scrambled exons generated by template switching or other artifacts of reverse transcription from a canonical linear RNA. In an analysis of publicly available RNA-Seq data from HeLa and H9 human embryonic stem cells, we found that RNAs with scrambled exons were enriched in non-poly-adenylated fractions. Taken together, these findings provide strong evidence that most scrambled exon sequences we detected in human RNA were derived from circular molecules.

While we have found that hundreds of human genes express circular RNA isoforms, the limitations of our experimental design may actually have led us to underestimate the prevalence of circular RNA isoforms. First, the size selection step during sequencing library preparation would miss small circular RNAs, or highly structured circular RNAs with fragmentation kinetics incompatible with our size selection. It is also possible that our size selection procedure would enrich for circular RNAs in the selected size range that failed to be fragmented. Second, although our sequencing depth was adequate to detect diagnostic junctional reads for many circular RNAs, it may not have been adequate to accurately identify and quantify rare circular isoforms, and our search for exon-exon junctions was restricted to exons annotated in RefSeq, which we know to be an incomplete catalogue of exon boundaries. Thus, we may still be underestimating the number or prevalence of circular RNAs in human cells.

**Figure 6:**
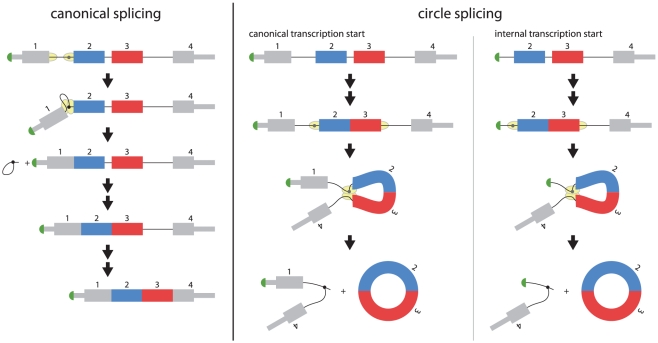
Models for generation of circular RNA. At left: a schematic diagram of the canonical splicing process splicing out the first intron of the a pre-mRNA of a 4 exon gene, and subsequent removal of introns 2 and 3. Canonical splicing of exon 1 to exon 2 occurs when the splicing machinery catalyzes the formation of the intron lariat and the attack of the free 3′ OH of exon 1 on the 3′ splice site upstream of exon 2. This produces a lariat containing intron 1 and a pre-mRNA with exons 1 and 2 spliced together. At right: a model for the production of circular transcripts. If there is a canonical transcriptional start, and if intron excision does not proceed sequentially in time from the 5′ to 3′ direction of the pre-mRNA, non-canonical pairing of 3′ and 5′ splice sites could be generated. Since the sequences of each 5′ splice site of the pre-mRNA contain the same splicing signals, it is possible that the 3′ splice site upstream of exon 2 is paired with the 5′ splice site downstream of exon 3 and splicing proceeds as if this 5′ splice site were paired with the 3′ splice site upstream of exon 4. In this case, exon 3 would be spliced upstream of exon 2, creating a pre-mRNA intermediate comprised of these two exons and intron 2. Canonical splicing would be predicted to excise this intron, leaving a circular RNA composed of exons 2 and 3. Non-canonical transcription start, as suggested in [25], could produce an orphan 3′ splice site corresponding to the first transcribed exon. This splice site could be paired with a downstream 5′ splice site, generating a circular RNA. In both models, the excised intron would be linear and branched, and expected to be quickly degraded.

The previously unappreciated abundance and diversity of circular RNAs in human cells raises important questions: What is the molecular mechanism of circular splicing? Recent evidence suggests that canonical pre-mRNA splicing does not necessarily proceed in sequential order from the 5′ to 3′ end of the RNA [24]. In this case, an orphan 3′ splice site upstream of the acceptor exon could serve as the acceptor site for a downstream 5′ splice site that is not paired with its canonical splicing acceptor, producing a circular transcript. Such a model is depicted in Figure 6. A particular example of this model, wherein an alternative promoter causes transcription initiation within the first intron, creates an orphan 3′ splice site that is later used by a downstream 5′ splice site could result in a circular RNA with exon 2 as the acceptor. This model would be consistent with our finding of enrichment of exon 2 as the acceptor exon and was suggested in [25].

How widespread and evolutionarily conserved is circular splicing? Our preliminary analysis of ribosomal-RNA depleted RNA from the mouse brain suggests there are hundreds of genes with scrambled isoforms in that organ; we would not be surprised to find that this phenomena is pervasive across the animal kingdom and perhaps more broadly. In addition, while we have not found evidence of differential circular isoform expression between tissues, this area should be further explored.

What functions do circular RNAs serve and what roles might they play in normal human biology or disease? The vast majority of circular RNA molecules we detected were transcribed from a gene that is also known to encode a conventional linear mRNA. *In vitro* studies have shown that circular RNAs can be translated, raising the possibility that some circular RNAs might encode proteins with functions distinct from those of their canonical counterparts [26]. A non-coding regulatory role is another distinct possibility.

Our data suggest there are at least a handful of known noncoding RNAs with circular isoforms. A circular isoform of the noncoding RNA ANRIL has been reported to correlate with INK4/ARF expression, as well as atherosclerosis risk, suggesting the possibility that circular RNAs might have some role in human disease. An antisense transcript of the CDR1 gene, relatively abundantly expressed in mouse brain, was recently shown to be circular, and to positively regulate the corresponding sense transcript [27]. A role in regulating the pool of RNA binding proteins or small RNAs capable of interacting with the conventional linear RNA counterpart is another possibility [28].

We cannot yet rule out the possibility that they are incidental to regulated splicing of a conventional linear RNA and perhaps accumulate due to their relative resistance to degradation. However, the high abundance of many circular RNA isoforms relative to their linear counterparts and lack of evidence for the predicted alternatively spliced linear RNA co-product suggest that circular RNA isoforms are not simply accidental byproducts of splicing. Further investigations of origins and activities of circular isoforms of mRNAs are likely to lead us in surprising directions.

## Methods

### Specimen Collection

Samples were collected following procedures approved by the IRB at Stanford University.

### RNA extraction from tissues

5 hyperdiploid acute lymphoblastic leukemia diagnostic bone marrow samples were obtained on a protocol approved by the Stanford Institutional Review Board. The cells were counted and frozen using Ficoll-Paque Plus (Amersham Biosciences, Piscataway, NJ) at a concentration of 0.5–10 million cells/ml. Peripheral blood was also obtained from the same patients after they were confirmed to be in remission based on the inability to detect minimal residual disease after induction therapy. The buffy coat was isolated by pipetting after centrifugation. CD19+, CD34+, and neutrophils that were isolated from the bone marrow of a single individual were purchased from All Cells Inc (Emeryville, CA). Total RNA was isolated from all samples using Trizol (Life Technologies, Carlsbad, CA) followed by RNEasy column (Qiagen, Valencia, CA) according to the manufacturer's instructions.

### RNA-Seq Library preparation

A modified protocol from our previous work was used to produce the sequencing libraries [21]. First, 2 or 5 ug of total RNA was depleted of ribosomal RNA using the ribo-zero kit (Epicentre, Madison, WI) from the normal hematopoietic and leukemia samples, respectively. RNA was then fragmented to about 350 bp using alkaline hydrolysis followed by first and second strand cDNA generation as previously outlined. cDNA was size-selected for a range between 300–500 bp before adaptor ligation to minimize the production of *in vitro* chimeric transcripts. A second size selection was then performed for 350–600 bp to select against chimeric inserts and remove unligated adapters. Libraries were PCR amplified for 15 or 20 cycles for the leukemia and normal hematopoietic samples, respectively. Concentrations were measured using the High Sensitivity DNA Kit for the Bioanalyzer (Agilent, Santa Clara, CA), and samples were then diluted to 10 pM. Clusters were generated using the Paired-End Cluster Generation Kit v2 with the cBot according to manufacturers instructions (Illumina, San Diego, CA). The samples were then sequenced on the Illumina Genome Analyzer II using kit v5. All samples were run as a single lane on a 80 bp paired-end run. Leukemia samples were sequenced together, while the remission blood and normal bone marrow subpopulations were a separate run. Sequencing data are deposited in GEO with series number GSE33772.

### Computational discovery of exon scrambling from RNA-Seq data

All mapping was performed using Bowtie version 0.12.1. Paired-end RNA-Seq data was mapped to the reference transcriptome (UCSC knowngene annotation) allowing up to three mismatches and any number of alignments. Reads uniquely aligning to the RefSeq annotation database were considered to represent gene expression. Reads failing the alignment to the UCSC knowngene annotation were mapped to a custom database of all intragenic exon-exon junctions in expressed genes with exons determined by the RefSeq database. Paired-end reads where read 1 mapped to an exon-exon junction inconsistent with the reference transcript and read 2 mapped to the same transcript, and were considered as supportive of an exon scramble. The total number of distinct paired offsets and mismatch profiles were summed, and the rate of reads supporting scrambled exons was computed per nucleotide (denoted j) and used as an estimate of the relative abundance of each scrambled isoform. Per-nucleotide gene expression (e) was estimated as the number of single reads mapping to a gene divided by gene length. The quantitative estimates of the relative expression of a scrambled transcript compared to the canonical linear isoform quoted in this paper was made using the ratio of j/e.

In a separate analysis, we employed paired-end mapping ratios to further quantify the relative abundance of scrambled isoforms to canonical linear isoforms. Each gene was tiled by dividing it into even length bins of 200 base pairs. For each pair of bins, the number of reads aligning to bin A in the+orientation and bin B in the - orientation was computed. To detect scrambled and canonical linear transcripts, bin A could be up or downstream of bin B. Conditional on reads aligning to bin A, the relative ratios of reads aligning to each bin B were computed. This procedure is further detailed in Text S1.

In addition to the data we generated, the following public data was downloaded from the SRA and analyzed: paired-end data from H9 cells (SRR065491, SRR065495, SRR065504, SRR065521, SRR066678, SRR066679) and HeLa (SRR065529) and single end data from [22] as described in the text.

### RT-PCR validation

Total RNA reaction was reverse transcribed using the SuperScript III First-Strand Synthesis System (Life Technologies, Carlsbad, CA) with random hexamers according to the manufacturer's instructions. 20 ng of cDNA was then used for each PCR validation using Platinum Blue PCR Supermix (Life Technologies, Carlsbad, CA). PCRs were run for 35 cycles under standard conditions with 30 second 55 degree extension.

### Statistical methods for computing the probability of observing evidence of a linear transcript under the tandem duplication hypothesis

The following procedure was used to calculate the per gene probabilities for observing evidence of a linear transcript among genes with evidence of scrambled exons.

1. Compute the empirical length distribution of all sequenced fragments by aligning reads to the RefSeq annotation.
2. Compute quantiles from 0–100 of empirical insert length distribution, and call this distribution F.
3. For each gene g with an exon X to exon Y (X≥Y) junction, compute inferred circle length, sum of length of exons between Y and X on a per gene basis (call this length L).
4. For each read 1 crossing the exon X- exon Y junction, approximate the probability that, if the scramble were contained in a linear transcript, read 2 would align to sequence upstream of exon y or downstream of exon X by 1-F(L). This is an approximation because the exact alignment offset of the junction (which can vary between 0 and 60) will influence this probability. Call this probability p. In most cases, this approximation will not be biased towards increasing or decreasing the calculated probability p. For n junctional sequences observed for a given gene, the probability of seeing 0 reads aligning to sequences outside of the exon X to exon Y interval is p_0_(g) = (F(L) )^n^.

Using the probabilities p_0_(g) over all genes, we computed the number of total genes expected to have observed reads supporting linearity under the hypothesis that scrambled exons are contained in linear, rather than circular, transcripts by computing the number of genes with p_0_(g) in the q^th^ quantile and multiplying this by the average p_0_(g) value. The result establishes an under-representation of reads supporting the hypothesis that scrambled exons are contained in transcripts with exons upstream of exon y or downstream of exon X.

### RNaseR assay

HeLa total RNA was isolated with TRIZOL, followed by rRNA depletion using RiboMinus (Life Technologies). Samples were then treated with DNase I (Fermentas, Glen Burnie, MD) for 15 min at 37°C, then diluted 16-fold into RNase R buffer containing 0, 3,10, or 100 units of RNase R (Epicentre) per µg of RNA. We have observed some batch to batch variation in the results of our RNase R assays and suspect that some batches of RNase R may have contaminating endoribonuclease activity: we include this as a note of caution. Reactions were purified with RNA Clean & Concentrator-5 columns (Zymo Research, Irvine, CA). Reaction product corresponding to 5 µg of input total RNA was reverse-transcribed in a 20 µl Superscript III reaction with random hexamers (Life Technologies). cDNA reaction (1/100^th^ of PCR volume) was used as template in a 35-cycle PCR with Phusion polymerase (New England Biolabs, Ipswich, MA), each cycle being: 94°C 5″; 68°C 15″; 72°C 2′.

### qRT-PCR on Cytoplasmic and Nuclear Fractionation

Total, nuclear, and cytoplasmic RNA were isolated using the PARIS Kit (Ambion, Austin, TX) according to the manufacturer's instructions. Primer and probe pairs for each qPCR reaction were designed using the RealTime PCR Assay Design Tool (Integrated DNA Technologies, San Jose, CA), see Table S7 for primer and probe pairs.

50 ng of cDNA was added to Taqman Gene Expression Master Mix (Applied Biosystems, Carlsbad, CA) along with primer and probe at final concentrations of 0.5 and 0.25 µM, respectively. Assays were run for 40 cycles using standard conditions on the 7900HT Fast Real-Time PCR System (Applied Biosystems, Carlsbad, CA). Ct values were generated using SDS software. Relative expression levels of linear and circular isoforms were computed as differences in Ct values, and standard errors were taken over biological replicates.

### Northern Blot

Probe was generated from PCR product of MAN1A2 circular RNA. A band that had previously been confirmed to correspond to the 5-2 junction by Sanger sequencing was isolated using the QUIAquick Gel Extraction Kit (Qiagen, Valencia, CA). The probe was then biotinylated using the Brightstar Psoralen-Biotin Kit (Ambion, Austin, Tx). Total and cytoplasmic RNA were isolated from HeLa using PARIS Kit (Ambion, Austin, TX). Labeled marker, 3.7 and 6.2 ug of total and ctyotplasmic RNA were run on a 1% agarose gel, transferred, and hybridized according to NorthernMax Kit protocol (Ambion, Austin, TX). 10 pM of probe was hybridized for 24–48 hours. Detection was performed using the BrightStar BioDetect Kit (Ambion, Austin, TX).

MAN1A2 Probe (481 bp)

CTATTCCCAACCTTGTAGGAATACGTGGTGGAGACCCAGAAGATAATGACATAAGAGAGAAAAGGGAAAAAATTAAAGAGATGATGAAACATGCTTGGGATAACTATAGGACATATGGGTGGGGACATAATGAACTCAGACCTATTGCAAGGAAAGGACACTCCCCTAACATATTTGGAAGTTCACAAATGGGTGCTACCATAGTAGATGCTTTGGATACCCTTTATATCATGGGACTTCATGATGAATTCCTAGATGGGCAAAGATGGATTGAAGACAACCTTGATTTCAGTGTGAATTCAGAGGTGTCTGTGTTTGAAGTCAACATTCGATTTATTGGAGGCCTACTTGCAGCATATTACCTATCAGGAGAGGAGGGAAGAGGAAGAACGTCTGAGAAATAAAATTCGAGCTGATCATGAGAAGGCCTTGGAAGAAGCAAAAGAAAAATTAAGAAAGTCAAGAGAGGAAATTCGAGCAG.

## Supporting Information

Figure S1 **Number of Genes with Circular Isoforms by Total Gene Expression and Detection Probability.** Total gene expression was computed for each of the three cell types (CD19, CD34 and neutrophils), and genes were ranked in expression according to sequencing reads per length. Expressed genes were categorized into 100 even quantiles by cell type. The number of genes with sequencing evidence of exon scrambling was computed for all genes in each quantile (each quantile contains the same number of genes) and are depicted as colored histograms. The median number of reads per length (gene expression) for each expression quantile determines the probability of detecting a scrambled transcript if it were expressed at the same rate as the median expression quantile. This detection probability is shown by a thin black line. If the probability of having scrambled exons did not vary by expression quantile (the uniform hypothesis), this probability would be proportional to the number of genes with scrambled transcripts. For all samples, the very highest expression quantile shows relative depletion in exon scrambling events compared to its expectation and compared to the number detected in the previous quantile under the uniform hypothesis.
(EPS)

Figure S2 **No-Template and No-RT Controls Do Not Produce PCR Products.** DNase-treated total RNA or cDNA from HeLa underwent 35 cycles of PCR amplification using primers specific for each of the noted scrambled transcripts. Each of the reactions with cDNA template produced expected products (C), while those without template (A) or with RNA that had not undergone reverse transcription (B) did not produce PCR products.
(EPS)

Figure S3 **Enrichment in Intron Length in Genes with Circular Isoforms.** We investigated whether genes with exon scrambles have typical intron lengths compared to the genome-wide distribution. At left: to test the association of intron length with scrambling events, we computed the median length of intron 4 (or, more generally, intron x) among scrambled isoforms where the donor exon was exon 3 (or, more generally, exon y), and found the relative quantile of this length in the length distribution of intron 4 in all human genes. We performed this analysis for all intron and exon pairs x and y where x,y≤9. At right: the analysis was repeated by stratifying scrambled junctions by acceptor exons. Several features of genes containing scrambled isoforms emerge from this analysis. First, the length of each intron of such genes are enriched compared to the whole-genome median, regardless of which donor or acceptor exon is involved in the scramble. This implies that genes with scrambled isoforms are longer than the typical gene. Secondly, there is an enrichment in the length of the intron immediately upstream of the acceptor exon (and to a lesser extent, in all upstream exons), and a relative depletion in the length of the intron immediately downstream of the acceptor exon (and to a lesser extent, in all downstream exons). A less striking enrichment in intron length is seen downstream of the donor exon.
(EPS)

Figure S4 **Denaturing RNA Gel Serves as Second Control for Cytoplasmic and Nuclear Fractionation.** 2 µg of total RNA from total lysate(T), as well as cytoplasmic (C) and nuclear (N) fractions were separated on 1% aagarose gel. As expected, the cytoplasmic fraction has higher concentrations of 28 s and 18 s ribosomal RNA bands. In addition, the nuclear fraction shows precursor ribosomal RNA bands that are absent in the cytoplasmic fraction.
(EPS)

Table S1 **List of scrambled isoforms detected in the leukemia data with RefSeq exon enumeration beginning at exon 0.**
(TAB)

Table S2 **List of scrambled isoforms detected in the leukocyte data with RefSeq exon enumeration beginning at exon 0.**
(TAB)

Table S3 **Sanger sequences and primers for RT-PCR validation (see Text S1 for more details).**
(DOC)

Table S4 **List of genes with reads in order inconsistent with the reference detected with RefSeq exon enumeration beginning at exon 0.** Table description and statistical methods for generating the table can be found in the Text S1.
(CSV)

Table S5 **Genome-wide assessment of evidence for scrambled isoforms being circular with RefSeq exon enumeration beginning at exon 0.** Table description and statistical methods for generating the table can be found in the Text S1.
(TAB)

Table S6 **Scrambled isoforms with evidence of being contained in linear RNA compiled from paired-end HeLa (poly-A selected), H9 (poly-A selected) and Leukoctye data with RefSeq exon enumeration beginning at exon 0 (see Text S1 for more details).**
(XLS)

Table S7 **qPCR primer and probe pairs.**
(DOC)

Table S8 **Table of scrambled exon-exon junctions detected from the public RNA-Seq dataset of ribosomal depleted mouse brain (see Text S1 for more details).**
(CSV)

Text S1 **Supplementary Methods.**
(DOC)
