# Supplementary material for: Circular RNAs Are the Predominant Transcript Isoform from Hundreds of Human Genes in Diverse Cell Types
Source: PLoS One. 2012 Feb 1;7(2):e30733. doi: 10.1371/journal.pone.0030733 (PMC3270023; doi:10.1371/journal.pone.0030733)
Supplement: Table S3 — Sanger sequences and primers for RT-PCR validation (see Text S1 for more details). (DOC) [file pone.0030733.s007.doc]

**Sanger Sequences and Primers**

>MAN1A2--3-2

CTATTCCCAACCTTGTAGGAATACGTGGTGGAGACCCAGAAGATAATGACATAAGAGAGAAAAGGGAAAAAATTAAAGAGATGATGAAACATGCTTGGGATAACTATAGGACATATGGGTGGGGACATAATGAACTCAGACCTATTGCAAGGAAAGGACACTCCCCTAACATATTTGGGAAGAGGAAGAACGTCTGAGAAATAAAATTCGAGCTGATCATGAGAAGGCCTTGGAAGAAGCAAAAGAAAAATTAAGAAAGTCAAGAGAGGAAATTCGAGCAG

>MAN1A2—4-2

CTATTCCCAACCTTGTAGGAATACGTGGTGGAGACCCAGAAGATAATGACATAAGAGAGAAAAGGGAAAAAATTAAAGAGATGATGAAACATGCTTGGGATAACTATAGGACATATGGGTGGGGACATAATGAACTCAGACCTATTGCAAGGAAAGGACACTCCCCTAACATATTTGGAAGTTCACAAATGGGTGCTACCATAGTAGATGCTTTGGATACCCTTTATATCATGGGACTTCATGATGAATTCCTAGATGGGCAAAGATGGATTGAAGACAACCTTGATTTCAGTGTGGGAAGAGGAAGAACGTCTGAGAAATAAAATTCGAGCTGATCATGAGAAGGCCTTGGAAGAAGCAAAAGAAAAATTAAGAAAGTCAAGAGAGGAAATTCGAGCAG

>MAN1A2—5-2

CTATTCCCAACCTTGTAGGAATACGTGGTGGAGACCCAGAAGATAATGACATAAGAGAGAAAAGGGAAAAAATTAAAGAGATGATGAAACATGCTTGGGATAACTATAGGACATATGGGTGGGGACATAATGAACTCAGACCTATTGCAAGGAAAGGACACTCCCCTAACATATTTGGAAGTTCACAAATGGGTGCTACCATAGTAGATGCTTTGGATACCCTTTATATCATGGGACTTCATGATGAATTCCTAGATGGGCAAAGATGGATTGAAGACAACCTTGATTTCAGTGTGAATTCAGAGGTGTCTGTGTTTGAAGTCAACATTCGATTTATTGGAGGCCTACTTGCAGCATATTACCTATCAGGAGAGGAGGGAAGAGGAAGAACGTCTGAGAAATAAAATTCGAGCTGATCATGAGAAGGCCTTGGAAGAAGCAAAAGAAAAATTAAGAAAGTCAAGAGAGGAAATTCGAGCAG

>MAN1A2—6-2

CTATTCCCAACCTTGTAGGAATACGTGGTGGAGACCCAGAAGATAATGACATAAGAGAGAAAAGGGAAAAAATTAAAGAGATGATGAAACATGCTTGGGATAACTATAGGACATATGGGTGGGGACATAATGAACTCAGACCTATTGCAAGGAAAGGACACTCCCCTAACATATTTGGAAGTTCACAAATGGGTGCTACCATAGTAGATGCTTTGGATACCCTTTATATCATGGGACTTCATGATGAATTCCTAGATGGGCAAAGATGGATTGAAGACAACCTTGATTTCAGTGTGAATTCAGAGGTGTCTGTGTTTGAAGTCAACATTCGATTTATTGGAGGCCTACTTGCAGCATATTACCTATCAGGAGAGGAGATATTCAAGATTAAAGCAGTGCAATTGGCTGAGAAACTCCTTCCTGCCTTTAACACACCTACTGGGATTCCTTGGGCAATGGTGAATTTGAAAAGGGAAGAGGAAGAACGTCTGAGAAATAAAATTCGAGCTGATCATGAGAAGGCCTTGGAAGAAGCAAAAGAAAAATTAAGAAAGTCAAGAGAGGAAATTCGAGCAG

>ZBTB46

GANGGCTCCCTGCTGTTCGAGTACCTGCCCAGAGGGGCCCACTCGCTGTCCCAGTCTGTAGAAGAGGCGACACCAGGGCTTCCAAATGAACAACCGAAAGGA

>ZNF346

CTTACCTGGGTGTNGGTGAGAGACATTGGTTCTTCTGGATCATGTGCTCCACTGGAAAGTCAGTGACAGCAGGGTCCGCCAGCCGCCCATAGCGTGCCCTTANTTTGAGCTTGGTCTCCTGTTTTCTGTGTTTCTTGCCCA

>ZFAND6

TTTTAAAGCCTTCTGTGTTTTACTGCAGCAGACTTCAGATTCATTCCCTGCACATCTTCTGTTTCAGGTACTGCTTTGTCCACAA

>ZBTB44

AATGTTTTCACACCCATCTTTTACTTCCTCTTCTACAGATGCTCTTCAAGGATGCAAATAAATCAGAAATGTCCTATAGAGGAAGACTGTGGACTAATCAGAAGGTCCTCTTGGACTTGTTCACTTCCTGGAACTGTCTGCTGATCACTCAGCGAACTCTGAGATGCACTGACAGGCTGGGACACT

>CHD1L

CACAGTATCTGCTGCTGTTAAGTTCATGCCAACTCCACTTTTTCTTATGTTCAGCCTCTTCCTTTTGCCTCTTCTTCTCCTCTATGAGTCTCCTTCTCTTGGCAGCTGCTTCA

>SFMBT2

GGGTCTTGCATATTGGAAGCTGACAAAGTGCTCTCCATGCCTGATGAGCAAGTGTCTCTT

CTCTTAATTCATCCTGCAGAAAAAATTACCTTAAACACTTCCATTGGAAGAGGAAATTTG

GCAGCATCAATAAGGGATTTTTCCAGAGTACATTTCCATTCAGAGGCCATCTTCAAAGGA

>FBXW7

ATGGGGAGGAGAGTTGGTAAACGGGCAGGTCCACAATACTACTGGAGTTCGTGACACTGTTAGTATGTGTATGTTCATCTTCTCTGCTACTATCATCAGACTGATCAAAATCGTCACTCTCCTGGTCCATCTCCTCCTCCTCCTCATCCTCCTCATCTTGTTCACCAGCATGTTCTTCATCTTCCTCTTGTTCTTCTTGGTTTCCTGAGGAGTCCTCATCTACCGAAATAAATCTATTATTGTTTTCTTCCAACTGTCCTTGCTGGGAATCATTTTGGCCTCCAGGTCTAGGTTCTACTCCAACAACTTCACCATTCCTTGCAGTGTGCTCCTCCTCTTGTTGTCTGAGTTGCTGTTGCTGTTCCTCCTCTACCACACGATTCATCTGTTCTTCATCTACCTGGCTTGAGGAAGGGTTACCTCTCAAAGAGCCTCCAGTTCGTCGTCTTTTGCTGCCCACAGAGAGCAGTTCCTGATTCATTTCCAAAAGCCAGCTTGCTACTTCTTGGACCTTGGCTAGACTATCAGAAGATGCAAAGGTTTTCACATTCCTCTGACCCAGTAACTCCACTTCTGGCAATCTATCCTAAGGAAGTAATCTTTTGTTGTTTTTGTATAGAATGGGGAGGAGAGTTGGTA

>CCDC126

CAGACATCAAAGCAGTGTCAAGTTACGTGAGCAAATACTAGACTTAAGCAAAAGATATGTTAAAGCTCTAGCAGAGGAAAATAAGAACACAGTGGATGTCGAGAACGGTGCTTCTATGGCAGGATATGAGTTAATAGAGTGGATACAACCTTGCTGAAGATGAAGAATATACAATATTGAGGATATTTTTTTCTTTTTTTTTTCAAGTCTTGATTTGGGGCTTACCTCAAGTTACCATTTTTCAGTCAAGTCTGTTTGTTTGCAA

>PCMTD1

TATCATTAAGTCATCATTATCTTCCCCAGCACTCACAGCTCCTCCCATGATAGTATTCAAATCAAATCATAAATTTAAAAGTGAAATAAAATTAGTAGAAATGGCTTCCAATATTGCACTTGATTTCCAAAAATAAATTAATCCTAAAATTAAGCCCACCATTGTACTTAAATATCCGGTTCCACTTCCCAGGTTAAGAAAAGACAATCCTGGTTGAAGTTTCAATGCTTCCATAACTTCAGAATAAATGCAAGGTGCTGACAAGTGA

>SETD2

AGAGTCAGCATCAGAGCAGCAGGAGGGAAAATGAAGAAGGAACGATCTCGTAAGAAGGATTCA

GAACACCTTTGTCCTAGAATATTGTGGAGAGGTACTCGATCATAAAGAGTTTAAAGCTCGAGTGAAGGAGTATGCACGAAACAAAAACATCCA

>SMARCA5

TCAGTGTTTATAACTTCGAAGGAGAAGACTATAGAGAAAAACAAAAGGGAGGCTTGTGGATCAGAATCTGAACAAAATTGGGAAAGATGAAATGCTTCAAATGATTAGACATGGAGCAACACATGTGTTTGCTTCAAAGGAAAGTGAGATCACTGATGAAGATATCGATGGTATTTTGGAAAGAGGTGCAAAGAA

> C1orf58 GAAGCATACGTTATTCAATGTCAGGCTGAAGCTCAAGAAGTATAACAGAAGATGAAGCAAAAGAAGTTCATCGAA

>FKBP8

GACGTCTGCAGATGTACGGTGACCACCTGGCCCTTGACCGGGCGGCTCGAACCTGGCGGCCCTGGGACCAGCGTCTTCTTCCTCAACAGCCCGTTCCCTGCCTTGGGGGCCGTAGCAGTACTTGGAGTCAGCAGTGACC

PRIMERS:

>MAN1A2—REVERSE

GTCAAGAGAGGAAATTCGAGCAG

>MAN1A2—FORWARD

CTATTCCCAACCTTGTAGGAATACG

>ZFAND6_4_ZFAND6_1-FWD;

TGTGGACAAAGCAGTACCTGA

>ZFAND6_4_ZFAND6_1-REV;

GCTTTCTGCCTTCCCTATTC

>ZNF346_4_ZNF346_1-FWD;

TGGGCAAGAAACACAGAAAA

>ZNF346_4_ZNF346_1-REV;

TGGTAATGTGCCAGCTTCTG

>ZBTB46_2_ZBTB46_1-FWD;

GCGCTCATGAGTAAGAACAGC

>ZBTB46_2_ZBTB46_1-REV;

CCTTTCGGTTGTTCATTTGG

>MAP2K4_2_MAP2K4_1-FWD;

TGGAGAAATTGGACGAGGAG

>MAP2K4_2_MAP2K4_1-REV;

CCTGTAGGATTGGGATTCAGA

>ZBTB44_1_ZBTB44_1-FWD;

AAGTGTCCCAGCCTGTCAGT

>ZBTB44_1_ZBTB44_1-REV;

AGCTTTCCAAGCATTTCCTG

>CHD1L_16_CHD1L_12-FWD;
GAAGCAGCTGCCAAGAGAAG

>CHD1L_16_CHD1L_12-REV;

GCTGCTTGCAAGTCATTCTG

> FBXW7--FWD

GAAGGAGGAAGGGAACCAAC

> FBXW7--REV

CCTCAGAACCATGGTCCAAC

> SFMBT2--3-1-FWD

GGTGGTGCACACAGAACAAC

> SFMBT2--3-1-REV

CTGAGCCGAGACACTTTTCC

>FKBP8_3-2-FWD;

TGGTCACTGCTGACTCCAAG;

>FKBP8_3-2-REV;

GACGTCACAGTCACCCAGAG;

>PCMTD1_1-1-FWD;

CACTTGTCAGCACCTTGCAT;

>PCMTD1_1-1-REV;

GGCTTGCTCCACTCTTTCAG;

>SCLT1_8-5-FWD;

TGATAATCGAACAACTCCGAAA;

>SCLT1_8-5-REV;

TGGCTAGCTGCAATTGTTCTT;

>SETD2_8-5-FWD;

AGAAATGTTTCTGCGGATCA;

>SETD2_8-5-REV;

GGATGTTTTTGTTTCGTGCAT;

>CCDC126_2-2-FWD;

TTGGACTCATTTGGGGATTG;

>CCDC126_2-2-REV;

GCAAACAAACAGACTTGACTGAA;

>SMARCA5_15-14-FWD;

TGGGCGAAAGTTCACTTAGAA;

>SMARCA5_15-14-REV;

TCTTTGCACCTCTTTCCAAAA;

>C1orf58_6_5-FWD

CAT CTC CCA AAA CTC ATT ACA CC

>C1orf58_6_5-REV

GCTGCAATCTTTAGGCTTCG

>FKBP8_3_2-FWD

TGGTCACTGCTGACTCCAAG

>FKBP8_3_2-REV

TCACCCAGAGTGAACACCAG
