# Supplementary material for: Circular RNAs Are the Predominant Transcript Isoform from Hundreds of Human Genes in Diverse Cell Types
Source: PLoS One. 2012 Feb 1;7(2):e30733. doi: 10.1371/journal.pone.0030733 (PMC3270023; doi:10.1371/journal.pone.0030733)
Supplement: Table S7 — qPCR primer and probe pairs. (DOC) [file pone.0030733.s011.doc]

**Scrambled Exon Probes Pairs**

MAN1A2 Scrambled

5'-/56-FAM/AGA CGT TCT TCC TCT TCC TCC TCT CC/36-TAMSp/-3'

1 5'-TCT GCT CGA ATT TCC TCT CTT G-3'

2 5'-TTA TTG GAG GCC TAC TTG CAG-3'

FFBXW4 5-2

5'-/56-FAM/TCC TTC ACT /ZEN/GGG ACA CTG GTC ATC T/3IABkFQ/-3'

5'-GTT CTG AGA CAC CTT CAC TCG-3'

5'-GAG TCT GGT CCA TTG CTA TCA G-3'

ZBTB44 2-2

5'-/56-FAM/ACT TCC TGG /ZEN/AAC TGT CTG CTG ATC AC/3IABkFQ/-3'

5'-ACA GAT GCT CTT CAA GGA TGC-3'

5'-GTC AGT GCA TCT CAG AGT TCG-3'

RNF220 2-2

5'-/56-FAM/CAA CCA GCA /ZEN/CTT TGG GTC AGG G/3IABkFQ/-3'

5'-CCA TCG GAG TCT CTT TCT GTG-3'

5'-CAG AGG ATC GGA ATG ACA GAG-3'

KIAA0182 2-2

5'-/56-FAM/AGA GCA TGA /ZEN/GCC ATG AGC CCA AG/3IABkFQ/-3'

5'-AAA GCA TCC CTA GCG AAG G-3'

5'-CAA GCT CGC CAA ACA GG-3'

CAMSAP1 3-2

5'-/56-FAM/TGT TGA TCC /ZEN/AGA ACA CCA TGG CAT CC/3IABkFQ/-3'

5'-CAA CGT AGA AAG GGT CTC TGA G-3'

5'-ATG ATC AGC ATC GAG AAG GTG-3'

**Canonical Probes Pairs**

MAN1A2

5'-/56-FAM/CAG TGC AAT TGG CTG AGA AAC TCC TT/36-TAMSp/-3'

5'-CAA GGA ATC CCA GTA GGT GTG-3'

5'-AGA GGT GTC TGT GTT TGA AGT C-3'

CAMSAP1

5'-/56-FAM/TC AGG CAG G/Zen/A TTC TGA CTC GGA TGT /3IABkFQ/-3'

5'-ATC CAC GGA TGG CTT CTT CCT TCA-3'

5'-AAT CGT GCT CGG CTT CCT CTA TGT-3'

RNF220

5'-/56-FAM/AC CGC TTT G/Zen/A GGA GTA TGA GTG GTG T/3IABkFQ/-3'

5'-ATG CTG TGG ACA TCG AGC ATG AGA-3'

5'-AGC ATC ACT GTC CGG GTT CTC TTT-3'

KIAA0182

5'-/56-FAM/TG AAT GAA C/Zen/T CAC CTT GAC GTC AAT GCA /3IABkFQ/-3'

5'-TGC ATG AAG CAA AGG ATT CCA GGC-3'

5'-TTG GTT GCT CGC TGA ATG ACA ACG-3'

FBXW4

5'-/56-FAM/GG TGC TGG A/Zen/T GTC ATG TAT GAG TCCC/3IABkFQ/-3'

5'-TGA CAC ACT TGG GCA GTG ACT TTC-3'

5'-ATA GCC ACA GGA CAG CAG TGT GAA-3'

ZBTB44

5'-/56-FAM/GA CTG TGT C/Zen/A TTG CCC GGA TCA GTT T/3IABkFQ/-3'

5'-ACC TGG TCT CCG AAT GTA ATG CCT -3'

5'-TGG CAG CAC GCC AAT ATA TGC AAG-3'

GAPDH

5' -/56-FAM/AG CCA CAT C/Zen/G CTC AGA CAC CAT GGG /3IABkFQ/-3'

5'-TCG ACA GTC AGC CGC ATC TTC TTT-3'

5'-ACC AAA TCC GTT GAC TCC GAC CTT-3'

XIST

5'-/56-FAM/AAA AGC AGG /ZEN/TAT CCG AAG CCC CG/3IABkFQ/-3'

5'-ATT AAA GCA GGT ATC CGA GGC-3'

5'-CAA CAC TCT GGC CCA TCG-3'
