# Supplementary material for: Circular RNAs Are the Predominant Transcript Isoform from Hundreds of Human Genes in Diverse Cell Types
Source: PLoS One. 2012 Feb 1;7(2):e30733. doi: 10.1371/journal.pone.0030733 (PMC3270023; doi:10.1371/journal.pone.0030733)
Supplement: Text S1 — Supplementary Methods. (DOC) [file pone.0030733.s013.doc]

**SUPPLEMENTAL METHODS:**

**Denaturing RNA Gel**

Fractionation was evaluated by running 2 μg of RNA from each fraction on a 1% agarose gel using MOPS buffer and NorthernMax Formaldehyde loading dye (Ambion, Austin, TX). Gel was stained using SYBR Safe (Life Technologies, Carlsbad, CA).

**Control RT-PCR**

12.5 µg HeLa total RNA was treated with 2 units DNase I at 37°C for 30', then cleaned up with Zymo RNA Clean & Concentrator-25 column (Zymo Research, Irvine, CA). 5 µg of DNase-treated RNA was used as template in a 20 µl Superscript III reaction with random hexamers, followed by RNase H digestion (Life Technologies, Carlsbad, CA). In addition to the standard reaction, control reactions were done omitting either RNA or Superscript. 0.2 µl of cDNA reaction was used as template in a 20 µl Phusion PCR (Thermo Scientific, Rockford, IL), for 35 cycles of [98°C 5"; 68°C 15"; 72°C 2']. 3 µl of PCR product was run on a 1.7% agarose gel.

**Methods to Infer Circular Transcripts are Enriched for Exon 2 as the Acceptor Exon**

We tested whether the pairs of exons (X,Y) resulting in scrambled exon X - exon Y junctions followed a uniform distribution (that is, conditional on the transcript containing n exons, observing each pair of exons producing an exon X- exon Y junction is equally likely). Under the uniform distribution, for a transcript with n exons, a junction between any pair of exons (X-Y) where Y<=X is equally likely, and the marginal likelihood that exon X is the donor exon involved in the junction is equal to X/(n+ n choose 2)= 2X/ (n^2+n). Enumerating exons from 0, and testing for exon scrambles involving exons 1,...,n[[1]](#footnote-2), the probability that exon Y is the acceptor exon involved in the junction is equal to (n-Y+1)/(n+ n choose 2)= 2(n-Y+1)/ (n^2+n). Using these null probabilities, collapsing across all genes with n exons, we computed the Pearson residuals for the deviation of observed frequencies at each (X,Y) pair from the expected frequencies, resulting in one residual for the exons X, Y among all transcripts with n exons. This statistic is denoted r_(X,Y,n). Since Pearson residuals are approximately normally distributed, the residuals computed from sets of genes with different exon numbers n can be compared. Summing the residual r_(X,Y,n) for the set of genes across n and Y or n and X results in a genome-wide test statistic for the representation of scrambling where the acceptor and donor exons are X and Y respectively. Under the null hypothesis, this value should be normally distributed with mean 0 and variance equal to 1/(M-X) where M is the maximum number of exons in the set of genes considered (in our analysis, we chose M=8 as the maximum). The most statistically significant test statistic observed for donor or acceptor exons was 3.17, corresponding to the second exon as the acceptor exon (in the RefSeq enumeration beginning with exon 0). Referred to the null N(0,1/8) distribution, this statistic was highly statistically significant and the most statistically significant enrichment observed.

**Statistical Enrichment of Long Introns Proximal to Scrambled Exons**

We further tested whether intron size upstream of the acceptor exon or downstream of the donor exon in scrambles was correlated with exon scrambling events. Long intron length has been proposed as a mechanism to produce scrambled transcripts by predisposing the transcript to alternative transcriptional starts within an intron. Other models predisposing exons adjacent to long introns to be circularized are diagramed in Figure 6. This would create an ‘orphan 3’ splice site upstream of the first exon (a general splicing process that could generate circular isoforms is diagramed in Figure 6). It has been suggested that this orphan splice site could later serve as an acceptor splice site for a downstream exon which would produce a circular transcript [1].

To test the association of intron length with scrambling events, we computed the median quantile of intron 3 (or, more generally, intron x) among isoforms where the donor exon was exon 3 (or, more generally, exon y), and found the relative quantile of this length in the quantile length distribution of intron 3 in all human genes. We performed this analysis for all intron and exon pairs x and y where x,y<=11. For intuition, this analysis uses exon enumeration beginning at exon 1. The result is depicted in Figure S1. Under the null hypothesis that there is no enrichment or depletion in intron sizes of genes stratified by having scrambled transcripts, quantiles are sampled uniformly on the interval [0,1]. Standard sampling theory for the median shows that the distribution of the difference between the sample median and .5 converges to a random variable with the N(0, 1/ (4n) ) distribution. The table of 95% CI for the sample median under the null hypothesis is shown in the table below. With the exception of the median size of intron 8 upstream of donor exon 9, the table combined with the figure legend shows that all intron lengths are statistically significantly enriched at a joint confidence level of .05 (correcting for multiple hypothesis testing).

Several features of genes containing scrambled isoforms emerge from this analysis. First, the length of each intron of such genes are enriched compared to the whole-genome median, regardless of which donor or acceptor exon is involved in the scramble. This implies that genes with scrambled isoforms are longer than the typical gene. Secondly, there is an enrichment in the length of the intron immediately upstream of the acceptor exon (and to a lesser extent, in all upstream exons), and a relative depletion in the length of the intron immediately downstream of the acceptor exon (and to a lesser extent, in all downstream exons). A less striking enrichment in intron length is seen downstream of the donor exon.

**Methods for Testing Differential Circular Isoform Usage Across Samples**

We tested whether, for a given gene, whether total read counts for a gene (based on the canonical linear annotation) were proportional to the number of paired-end reads directly supporting scrambled exons (junctional counts). For each of the three leukocyte cell types, we formed a 3 by 2 contingency table: the rows represented the three cell types, and the columns contained the total gene counts and junctional counts, respectively, corresponding to that cell type. A hypergeometric test of independence of rows and columns was performed. Under the null hypothesis, circular isoform expression is proportional to total gene expression, and no statistically significant deviation of of proportionality between the two columns should be observed. Our evaluation showed no genome-wide statistically significant deviation of the hypergeometric test statistics from the null hypothesis (as evaluated by the FDR). This lack of significance could be due to a real biological phenomenon where circular isoforms in leukocytes cell populations are generated at a rate proportional to transcription, or due to a lack of statistical power because of low sequencing depth.

**Methods for Inferring the Number of Circular Isoforms Based on Sampling Depth**

For each gene expression quantile (Supplemental Figure 1), we calculated the the probability of detecting a circular transcript if it were expressed at the same level as our estimate of gene expression based on the median of reads per transcript length for genes in that quantile. In order to find a read supporting a scrambled exon-exon junction, a sequencing read must align +/- 30bp of the scrambled junction. To illustrate our method of calculation, if the median gene expression in a particular quantile is 1/60 reads per length, the probability of not seeing any reads supporting (failing to detect) a scrambled junction is (1-1/60)^60, or approximately, exp(-1). The probability of detecting a scrambled is prob(detection) = 1-prob(no detection) = 1- (1-expected reads in a 60 bp window / 60)^60= 1-exp(-EXPECTED READS PER 60)= 1- exp(-60*readsperbase).

**Description of Fields in Supplemental Tables 1-6 and 8**

Note: genes with NR_* prefixes correspond to RNA annotated as non-coding.

Table S1

List of scrambled isoforms detected in the leukemia data by common gene name and scrambled exon pair.

Table S2

List of scrambled isoforms detected in the leukocyte data by common gene name

and scrambled exon pair.

Table S3

Sanger sequences and corresponding primers used for RT-PCR validation of scrambled exon pairs. Breakpoints that represent exon scrambling are denoted by underlined sequence. With the exception of MAN1A2, all sequences were derived from directly sequencing PCR product. Products from MAN1A2 were obtained by TOPO cloning.

Table S4

Statistical evaluation of pairs of reads where both sides mapped entirely to the canonical linear transcript but in orientation inconsistent with the genomic reference. This analysis was performed by binning each canonical transcript into bins of length roughly 200 (call these bins b_1, b_2, … in increasing order from the 5' to 3' end of the canonical linear transcript). Conditional on a read having side 1 mapping in the + orientation to bin b_i, b_j was found where b_j had the the maximum number of of reads from side 2 among all b_j with j<=i ( cases where j=i were subsequently disregarded from the analysis but are included in our table). This bin represents the bin containing the maximum number of reversed reads. Conditional on a read having side 1 mapping in the + orientation to b_i, b_k was found where b_k had the the maximum number of of reads were side 2 and k>i. This bin represents the bin containing the maximum number of wildtype reads. The exon numbers containing the endpoints of b_i, b_j, b_k were determined. This enumeration used the standard RefSeq enumeration of exons beginning with exon 0. We modeled the probability (p_i) that, conditional on a read aligning in the + orientation in the i^th^ bin, the read would fall in b_j compared to the probability it would fall in b_j or b_k. p_i was computed using a binomial estimator and the 95% binomial confidence interval was also computed.

This analysis is summarized in Table S4. Table descriptions are as follows, given through examples of the first row of the table in parentheses:

1. NM identifier: (NM_000519)
2. common gene name (HBD)
3. sequencing lane (lane6)
4. left side bin (b_i) nucleotide start site (0)
5. left side bin (b_i) nucleotide stop site (199.02)
6. right side bin (b_k) nucleotide start site: (398.05)
7. right side bin (b_k) nucleotide stop site: (597.08)
8. right side bin (b_j) nucleotide start site (0)
9. right side bin (b_j) nucleotide stop site(199.02)
10. statistical method to compute binomial probability and confidence interval (exact)
11. number of reads in b_j (1)
12. number of reads in b_k (19)
13. p_i (0.052)
14. lower 95% confidence interval (0.001)
15. upper 95% confidence interval (0.260)
16. exon position of left side bin (b_i) nucleotide start site (0)
17. exon position of left side bin (b_i) nucleotide stop site (0)
18. exon position of left side bin (b_k) nucleotide start site (1)
19. left side bin (b_k) nucleotide stop site (2)
20. exon position of left side bin (b_j) nucleotide start site (0)
21. left side bin (b_j) nucleotide stop site (0)

Table S5

Genome-wide assessment of evidence for scrambled isoforms being circular. Each read is a separate entry in the table. Sequencing lanes 1-3 correspond to samples CD19, CD34 and neutrophil, respectively. As described in the main text, we computed the density of paired-end reads directly supporting each scrambled exon junction to the density of paired-end reads mapping to the entire transcript. This ratio, as a percent, is defined as junction density percent (JDP). Note that the point estimate JDP has large variance when the total junction counts are small. Also, as described in the main text, conditional on one of two paired-end reads (side 1) aligning to a scrambled exon X - exon Y junction, the total size of exons Y, Y+1, … X gives the size of a predicted circle containing exons Y+1, …, X. Evidence for a linear transcript can be obtained when side 2 maps outside of the predicted exons involved in the circle. We computed the probability of this event, using the cumulative distribution of the empirical insert length distribution, for each such read. These statistics, along with other information about the isoform is given in Table S5 and described below through examples with the first row in the table.

1. junctional density, JDP, (80)
2. total junctional counts for the scrambled isoform (1)
3. sequencing lane in which this isoform was detected (lane1)
4. common gene name (FAM125B)
5. donor exon (6)
6. acceptor exon (1)
7. NM identifier (NM_033446)
8. orientation of read aligning to junction (-)
9. start of read aligning to linear NM transcript in NM coordinates (588)
10. strand of read aligning to linear NM transcript in NM coordinates (+)
11. probability of paired-end read falling in an exon Z which is interior to the predicted circle. Z is defined as acceptor exon [in this case 1] <= Z <= donor exon [in this case 6]. The computed probability is based on cumulative distribution of the empirical insert length distribution (0.99)
12. insert size required for paired-end read falling outside of exons 1-6 (700)
13. inferred circle length--should be rounded to nearest nucleotide (675.99)

Table S6

Scrambled isoforms with evidence of being contained in linear RNA compiled from paired-end HeLa (poly-A selected), H9 (poly-A selected) and leukoctye data. When one of two paired-end reads (side 1) aligns to a scrambled exon X - exon Y junction, evidence for a linear transcript can be obtained when side 2 maps outside of the exons predicted to be involved in the circle. Summaries of such reads supporting linear transcripts are given in Table S6. The 4 fields of the table are as follow: common gene name, exon X, exon Y and total distinct counts. As mentioned in the main text, IFI16 is a positive control in this experiment and was detected in 2/3 samples. This method can produce false positive evidence for linearity when exons are homologous, and we have not undertaken a statistical assessment of this evidence.

Table S8

Table of scrambled exon-exon junctions detected from the public RNA-Seq dataset of ribosomal depleted mouse brain.

Fields:

1. readid: id of read in sequencing data supporting scramble
2. junction_strand: strand on which read supporting junction aligns,
3. exon1: upstream exon in scramble
4. exon2: downstream exon in scramble,
5. junction_start: offset of junctional read to exon-exon database
6. junction_seq: sequence of junctional read,
7. junction_read read (1 or 2) aligning to the junction,
8. junction_mismatch: number of mismatches of the junctional read
9. read_mismatch number of mismatches of the read aligning to the reference transcript
10. readstrand: strand of read aligning to the reference transcript
11. nm_id: nm identifier of transcript
12. readstart: start of read aligning to the reference transcript
13. readseq: sequence of read aligning to the reference transcript
14. genename:common gene name of transcript

**Reference**

1. Braun S, Domdey H, Wiebauer K (1996) Inverse splicing of a discontinuous pre-mRNA intron generates a circular exon in HeLa cell nuclear extract. Nucleic acids research 24: 4152-4157.

1. This convention is both for mathematical convenience and the convention used by the RefSeq and UCSC knowngene annotations. [↑](#footnote-ref-2)
